# From PHEIC to PHECs: reclaiming Africa’s agency in global health security governance

**DOI:** 10.1186/s12992-025-01177-6

**Published:** 2026-02-04

**Authors:** Nelson Aghogho Evaborhene

**Affiliations:** PandemiX- Centre for Interdisciplinary Study of Pandemic Signatures, Department of Social Science and Business, Roskilde University, Roskilde, 4000 Denmark

**Keywords:** Africa CDC, Public health emergency of continental security, Decolonial health governance, Pan-Africanism, Global health security

## Abstract

In the wake of COVID-19 pandemic, the African Union elevated the Africa Centres for Disease Control and Prevention (Africa CDC) to autonomous status, empowering it to declare Public Health Emergencies of Continental Concern (PHECs). This mechanism was first operationalized in 2024 in response to sustained mpox transmission across multiple African countries, despite the World Health Organization’s (WHO) earlier lifting of the Public Health Emergency of International Concern (PHEIC). This article examines the PHECs as a decolonial intervention in global health governance. Applying the Critique, Reform, Withdrawal, and Transformation (CRWT) framework, I argue that the PHECs reflect both a strategic withdrawal from overreliance on the WHO PHEIC system and a transformative effort to embed African-led governance rooted in Pan-African solidarity. The article highlights mechanisms for sustaining Pandemic Prevention, Preparedness, and Response (PPPR), including tiered activation, cross-sectoral oversight, civil society engagement, and alignment with continental financial instruments. Invariably, the PHECs represent a critical reconfiguration of Africa’s role in global health—from recipient of external interventions to architect of regional norms, practices, and accountability. Its promise lies not in rejecting multilateralism but in recalibrating it, embedding regional expertise, political leadership, and operational autonomy within broader global frameworks.

## Introduction

The COVID-19 pandemic exposed the profound limitations and inequalities of global health governance, particularly for countries in the Global South [1]. Amid social economic challenges, supply chain failures, and uneven vaccine distribution, the African continent was once again placed at the receiving end of global decisions that did not always reflect its priorities or context [2]. Against this backdrop, the Africa Centres for Disease Control and Prevention (Africa CDC) has emerged as a critical institution reshaping the continent’s approach to public health emergencies.

Through cross-continental coordination, pooled procurement platforms, and high-level diplomatic engagement, it helped secure vaccines, diagnostics, and therapeutics at a time when many African countries faced exclusion from global markets. The COVID-19 crisis demonstrated that Africa CDC could broker collective action among Member States, mobilize critical resources, and contribute to shaping emerging international norms rooted in equity [3, 4].

Recognizing its growing achievement, the African Union (AU) formally elevated the Africa CDC to an autonomous institutional entity in 2022 [5]. This landmark decision endowed the nascent institution with enhanced authority, including the capacity to independently declare Public Health Emergencies of Continental Security (PHECs)—a mechanism designed to coordinate continent-wide responses to emerging health threats.

Africa CDC exercised this authority for the first time in August 2024, following sustained Mpox transmission across multiple countries [6]. The declaration has been described as “a major step in driving regional ownership,” [6] marking a pivotal shift in how the continent asserts its agency and leadership in global public health governance.

While modest in institutional form, the PHECs mechanism embodies a profound reconfiguration of power: it represents both a symbolic and practical assertion of continental autonomy. This commentary argues that the PHECs should be understood as a strategic act of withdrawal and transformation within a broader decolonial project—one that not only critiques global health inequities but builds new, African-led governance systems rooted in collective action and Pan-African solidarity.

## Why the PHECs matters

The establishment of the PHECs by Africa CDC is a pivotal development in the landscape of global health governance, particularly for the continent. Traditionally, the World Health Organisation (WHO) Public Health Emergency of Continental Security (PHEIC) mechanism—central to the *International Health Regulations (2005)* (IHR)—has long served as the primary global alert system for serious health threats [7]. A PHEIC is declared when a disease outbreak or biologic threat is deemed extraordinary, poses a public health risk to other states through international spread, and requires a coordinated international response. When at least two of these conditions are met, states are required to notify WHO under the IHR.

Once a potential PHEIC arises therefore, the WHO Secretariat assesses the situation and convenes the *Emergency Committee*—an independent panel of global experts. The Committee then reviews all available evidence, including epidemiological data, cross-border transmission risks, and the adequacy of national responses. Based on this assessment, it advises the Director-General on whether the event meets the criteria for a PHEIC and issue, where appropriate, *Temporary Recommendations* to guide Member States’ actions.

Although the Emergency Committee’s advisory function is designed to safeguard scientific independence, its composition, deliberative processes, and application of criteria have frequently attracted scrutiny. Critics point to inconsistencies in interpretation, limited transparency in proceedings, and the potential influence of political considerations in shaping recommendations [8–11]. Thus, “determining what constitutes a PHEIC is a highly complex decision process that cannot be reduced to public health factors and epidemiological trends” [9]. Ultimately, however, the final authority to determine and declare a PHEIC rests with the Director-General. Once declared, such a determination activates both normative and operational mechanisms, including the mobilization of technical teams, release of emergency funds, coordination of international surveillance and response efforts, and deployment of medical countermeasures such as vaccines.

Since the IHR’s amendment, eight PHEIC have been declared, each illustrating both the strengths and limitations of the mechanism [11]. H1N1 influenza (2009) showcased rapid mobilization but drew criticism for overreaction. Polio (2014–ongoing) highlighted challenges of long-term declarations. Ebola outbreaks in West Africa (2014–2016) and the DRC (2019) revealed delays due to bureaucratic caution, political sensitivities, and tensions with national sovereignty. Zika virus (2016) underscored difficulties in balancing evidence-based risk with urgent global concern, and COVID-19 (2020) tested WHO’s coordination under unprecedented political scrutiny.

However, the monkeypox PHEIC (2022) was particularly notable: for the first time, the WHO Director-General declared a PHEIC despite the Emergency Committee not recommending it [12]. As Wenham and Eccleston-Turner observe, this decision reflected WHO’s attempt to reassert its authority in global disease governance following the erosion of institutional trust during the COVID-19 pandemic [13]. By exercising independent judgment, WHO not only reaffirmed its formal mandate but also demonstrated its willingness to act decisively amid expert disagreement. The episode underscores the normative and political dimensions of global health decision-making—revealing how WHO’s authority both shapes and is simultaneously shaped by broader international power dynamics. For Africa, such delays have had tangible consequences: outbreaks spread, mortality rises, health systems are overwhelmed, and local expertise is sidelined.

Africa CDC’s subsequent declaration of Mpox as a PHEC in August 2024 reflected sustained regional health concerns despite global de-escalation. By that time, 17,541 cases and 517 deaths had been reported across 13 AU member states, representing a 160% increase in cases and a 19% increase in deaths in 2024 compared with the same period in 2023 [6], with the Democratic Republic of the Congo accounting for the majority of cases and deaths. Later that month, following the Africa CDC PHECs, WHO reinstated the PHEIC for Mpox, signaling renewed international attention.

While it remains unclear whether Africa CDC’s action directly influenced WHO’s decision, this timing raises an important question: can regional leadership now shape global health agendas, rather than merely react to them? This sequence illustrates how the PHECS can spotlight regional health dynamics and inform broader global responses, particularly when disease burden does not align neatly with global epidemiological thresholds.

The contrast between WHO’s PHEIC and Africa CDC’s PHECs is summarized in Table 1, highlighting differences in authority, decision-making speed, representation, scope, triggers for action, and integration with global frameworks.

**Table 1:** Comparative dimensions of who’s PHEIC and Africa CDC ’s PHECs mechanisms

Dimension	WHO PHEIC	Africa CDC PHECs	Implication
Authority & Legitimacy	Centralised global authority	Regional African authority	PHECS asserts regional sovereignty within a multilateral system
Decision-making Speed	Maybe delayed due to political negotiation	Faster and more context-specific	Enables timely African response to emerging threats
Representation & Participation	Dominated by Global North leadership	Led by African experts and institutions	Shifts epistemic authority to the continent
Scope of Declaration	Global/international impact required	Continental (AU member states) focus	Tailored to Africa’s specific health threats and political structures
Trigger for Action	Requires significant cross-border international spread	Based on regional threat to AU-wide public health security	Enhances responsiveness to regional epidemics
Recognition and Integration	Automatically triggers international attention, funding mechanisms	Requires advocacy for international recognition	PHECs must be institutionally validated in global frameworks
Relation to Multilateralism	Represents global health solidarity, but can reflect power imbalances	Embeds Pan-African solidarity within global health architecture	PHECs complements, not rejects, multilateralism

## The Africa CDC PHECs as a decolonial project: the CRWT framework

To fully grasp the significance of the PHECs mechanism, it is useful to analyze it through the lens of the CRWT framework—a conceptual model that delineates four strategic pathways through which formerly colonized, or structurally marginalized actors engage in the process of decolonization or exercise agency within conditions of constraint. These pathways are Critique, Reform, Withdrawal, and Transformation. These pathways were mapped using pan-african thoughts and ideas (see Table 2). The table below outlines each strategy with relevant global health examples and demonstrates how Africa CDC’s PHECs mechanism reflects these approaches:

- **Critique** entails the analytical exposure of entrenched structural inequalities, colonial legacies, and power imbalances within global systems. It includes the identification of limitations and exclusions that can arise in traditional global health governance, such as Eurocentric or donor-driven frameworks that have historically marginalized African expertise and leadership. For example, scholars have critically examined delayed PHEIC declarations during past crises (e.g., Ebola in 2014–2016) [8, 9] and raised concerns about the discontinuation of the mpox PHEIC in 2023, despite sustained transmission in African countries [12].
- **Reform** involves efforts to address these inequities from within existing global institutions, including calls for increased representation, improved responsiveness, and more equitable resource allocation. Reforms implemented after the West African Ebola epidemic—such as the creation of WHO Emergency Committees [14], amendments to the IHR, and the establishment of the Contingency Fund [15]—have led to some progress. More recently, states have adopted the pandemic treaty. However, they have not always resulted in the structural shifts necessary to fully redress regional disparities [16].
- **Withdrawal** represents a strategic disengagement—either partial or targeted—from global mechanisms perceived to perpetuate dependency or overlook regional priorities. Crucially, this does not imply isolationism, but rather a recalibration that creates space for independent decision-making. Africa CDC’s development of the PHECs mechanism and the establishment of the Africa Epidemics Fund illustrate this pathway, enabling the continent to define and finance public health emergencies through African-led platforms.
- **Transformation** entails the creation of new institutions, systems, and governance structures that are rooted in local political, cultural, and epistemic foundations. These initiatives reflect a long-term strategy of institutional autonomy. The elevation of Africa CDC to autonomous status within the African Union [5], and its growing leadership in orchestrating continent-wide public health responses, embodies this strategic transformation.

Using the CRWT framework, Africa CDC’s implementation of the PHECs mechanism demonstrates a calculated blend of withdrawal from inherited hierarchies and transformation of the global health governance landscape. This is not a retreat from multilateral cooperation, but a strategic recasting of relationships, authority, and responsibility. Through the PHECs, the continent asserts the right to define health security on its own terms, anchored in institutions that reflect its specific epidemiological realities, political aspirations, and economic constraints.

In doing so, Africa CDC advances a model of engagement that neither rejects multilateralism nor remains subordinate to it, but, instead reshapes the terms of participation in ways that enhance continental agency and collective autonomy.

**Table 2:** Applying the CRWT framework to the Africa CDC’s PHECs mechanism

CRWT Strategy	Description	Global Health Example	Relevance to Africa CDC’s PHECs
Critique	Analytical exposure of structural inequalities and colonial legacies embedded in global health governance	Criticism of WHO’s delayed PHEIC declaration during the 2014–2016 Ebola outbreak; decision to end mpox PHEIC in 2023 despite ongoing African cases	Highlights limitations of centralized decision-making and underrepresentation of African realities
Reform	Pursuit of changes within existing institutions to improve equity, representation, and responsiveness	Establishment of the WHO Emergency Committee, Contingency Fund post-Ebola, IHR amendments, and pandemic treaty negotiations	Signals recognition of deficiencies in the global system, but has delivered only incremental improvements
Withdrawal	Strategic disengagement from overreliance on dominant systems to assert regional autonomy	Africa CDC launching the PHECs mechanism in parallel to WHO’s PHEIC; creation of the Africa Epidemics Fund	Enables Africa to declare and fund emergencies independently, reducing dependence on external donor-driven mechanisms
Transformation	Building new, self-defined institutions rooted in local governance, values, and needs	Elevation of Africa CDC to an autonomous AU entity; institutional growth post-Ebola; creation of the Africa Epidemics Fund	Embodies the shift toward African-led, financially sovereign, and context-responsive public health governance mechanisms

## Pan-Africanism, health security, and the PHECs mechanism

In global health governance, neoliberalism functions as a deep structural logic that shapes what forms of action, discourse, and institutional design are deemed legitimate [17]. It prioritises market efficiency, individual responsibility, and technocratic managerialism over structural justice. This orientation can be traced from the era of World Bank structural adjustment programmes to the so-called “golden age” of global health in the 2000s [18], marked by the rise of public private partnerships that ultimately shaped the global COVID-19 response [19]. Within this paradigm, biomedicine and neoliberalism converge into biopower, producing a regime in which risk, behaviour, and even sexuality become individualised and governable through surveillance systems, metrics, and behavioural regulation. Neoliberal legality has therefore not only influenced but consistently structured global health policy decision making.

The establishment and operationalisation of the PHECs mechanism must be situated against this prevailing orthodoxy and understood through the lens of a contrasting intellectual tradition: Pan Africanism. As a political and ideological movement grounded in unity, solidarity, and collective self-reliance, Pan Africanism has evolved from its mid twentieth century focus on anti-colonial struggle and political independence into a contemporary project of continental integration [20]. Despite this evolution, it remains the normative foundation of Africa’s collective governance architecture. As Abrahamsen and colleagues observe, Pan Africanism informs and constrains the political practices of the African Union and continues to shape how the continent positions itself within global affairs [21].

Within the institutional framework of the AU, Pan-Africanism has expanded to encompass health and development challenges. Article 3 of the AU’s Constitutive Act explicitly mandates the promotion of common positions on health and development challenges, including the eradication of preventable diseases [22]. Africa CDC emerged from this Pan-African spirit in the aftermath of the 2014–2016 Ebola crisis, with the explicit aim of strengthening the continent’s collective health security infrastructure.

During the **COVID-19 pandemic**, this vision was translated into practice as the AU—through Africa CDC—coordinated continent-wide surveillance, pooled procurement of vaccines, diagnostics, and therapeutics, and harmonized response strategies under a unified continental framework [3]. This was reflected in initiatives such as the Africa Medical Supplies Platform, the African Vaccine Acquisition Task Team, and the Partnership for African Vaccine Manufacturing [23]. The emergence of newer continental institutions such as the Africa Medicines Agency and the Africa Pharmaceutical Technology Foundation further consolidates this architecture of collective health security.

The PHEC mechanism thus joins this lineage as a distinctly continental tool for collective action. Article 3, paragraphs (e) and (f), of the Africa CDC Statutes approved by AU Heads of State in July 2022 [24], provides its legal foundation: empowering Africa CDC to declare PHECs in consultation with affected Member States and to coordinate health emergency responses, particularly those declared as PHECs or PHEICs. According to the Africa CDC Director General, the Mpox PHEC declaration followed extensive consultation across the AU system, involving Member States, technical experts, and regional institutions, ensuring that the decision reflected both scientific evidence and political consensus [25].

In his announcement of the PHECs, the DG described it as a *“clarion call to action*,*”* affirming that the continent could “no longer afford to be reactive.” [25] He invoked the principle of *One Africa*—resilient, resourceful, and resolute—underscoring the ongoing burden of disease in the continent despite global attention waning after the 2022–2023 WHO Mpox PHEIC. Similarly, President Cyril Ramaphosa, as AU Champion on Pandemic Prevention, Preparedness, and Response, framed the decision as both corrective and emancipatory, emphasizing equitable access to vaccines, therapeutics, and diagnostics, alongside the accelerated operationalization of the African Epidemics Fund [26]. The subsequent disbursement of USD 10.4 million by the AU Permanent Representatives Committee [27], followed by the Africa Epidemic Fund’s activation in February 2025, and a tripartite agreement with the EU’s Health Emergency Preparedness and Response Authority and Bavarian Nordic to distribute 200,000 vaccine doses, demonstrated the PHEC’s capacity to mobilise resources rapidly.

While these narratives demonstrate pan-African solidarity, the PHEC is also reinforced by structural imperatives. A neorealist perspective makes this explicit by underscoring the constraints that shape the continent’s health governance [28]. For instance, COVID-19 exposed the continent’s vulnerability to vaccine nationalism, export controls, and marginalization in global decision-making, revealing the fragility of multilateral solidarity. In this context, the PHEC mechanism is a strategic tool through which African countries enhance bargaining power, mitigate risk, and assert autonomy within an anarchic global health order. From this structural vantage point, continental unity becomes not only an ideological commitment but a strategic necessity.

Together, these developments translate Pan-African principles—collective decision-making, shared responsibility, and self-determination—into institutional practice. Yet, as Africa CDC Director General Jean Kaseya clarified, this model of autonomy is not one of isolation [25]. Rather, Africa CDC continues to collaborate with WHO, UNICEF, bilateral and multilateral agencies, the private sector, and academia, deploying experts, strengthening laboratories, and enhancing cross-border surveillance. The PHECs mechanism therefore represents both a normative and structural recalibration of the continent’s engagement in global health, positioning the Africa CDC as a coordinated actor capable of defending continental interests within an inequitable international system.

## Implications of the PHECS for global health security governance

While the PHECs projects a distinctly Pan-African vision of collective action, it simultaneously reflects enduring continuities with the prevailing logic of global health security [29]. Both paradigms prioritize containment at the source, intensified surveillance, and biomedical countermeasures—particularly vaccine deployment—as primary strategies for managing transnational risk. This convergence exposes a structural paradox: the PHECs mechanism diverges from the global system in governance philosophy and locus of authority, yet remains tethered to the same colonial, neoliberal, and securitized rationality that equates health security with control, quantification, and containment.

The implications of this duality are multifaceted. Alignment with global health security norms enhances Africa CDC’s interoperability, legitimacy, and access to technical and financial resources, strengthening its capacity to coordinate with international actors. Conversely, reliance on global frameworks risks constraining Africa CDC normative authority, reproducing hierarchies of expertise, and perpetuating dependency in critical areas such as biomedical manufacturing and surveillance infrastructure.

The fragility of this dependency becomes even more pronounced during periods of global scarcity. A continental emergency declaration heightens demand for vaccines and therapeutics as seen with Mpox, yet access remains contingent on decisions made by external manufacturers and governments that traditionally prioritize domestic needs. In this case, restriction of export and vaccine nationalism, already familiar from the COVID-19 response, could leave African countries unable to operationalize the very mechanism designed to enhance continental security.

A historical parallel [9] can be seen in the 2016 yellow fever outbreak in Angola and the DRC. Although a PHEIC could have been declared under the formal criteria [11], doing so risked triggering vaccine nationalism, with countries hoarding supplies and creating shortages for affected nations. Empirical evidence suggest that the risk of inequitable access influenced the Emergency Committee’s decision-making [30].Without robust manufacturing capacity, financing and guaranteed access provisions therefore, the PHECS mechanism risks not only perpetuating biopolitical control but could also unintentionally entrench the very inequities it aims to address.

This paradox of expanding African agency within inherited hierarchy therefore raises a deeper question: what does health security mean for the continent, and who has the authority to define it? Much of the global health security agenda remains oriented toward preventing outbreaks from spreading across borders, a framing that aligns with donor priorities and risks marginalising the continent’s wider health needs and structural determinants of vulnerability.

Despite these tensions, the Joint Action Plan for Mpox between WHO and Africa CDC marked a deliberate move toward institutional co-leadership [31]. Structured around ten operational pillars, Africa CDC assumes leadership over surveillance, laboratory systems, vaccination, and logistics, while WHO retains oversight of infection prevention, biosafety, and behavioral insights. Although material asymmetries in financing and supply chains persist, embedding continental institutions such as the African Medicines Agency, AUDA-NEPAD, and Afreximbank within the joint mechanism signals a deeper integration of African institutional capacity into the global health architecture.

This deepening collaboration, however, surfaces critical questions for the future of global health governance. Will WHO and other international actors, including private stakeholders, formally recognise regional mechanisms such as the PHECs as legitimate authority? Will donors and partners adapt funding and engagement models to support regional autonomy rather than defaulting to global institutions? These dynamics underscore key fault lines in the ongoing project of global health decolonization, pointing toward an evolving balance between multilateral cooperation and continental sovereignty.

To realize its transformative potential therefore, the PHECs framework must evolve beyond replication of global health security logics toward a distinctly Pan-African epistemology of health governance. Disease outbreaks do not occur in a vacuum; they are shaped by histories of inequality, extractive political economies, ecological disruption, and the enduring legacies of colonial power that structure vulnerability across the continent [32]. Addressing these determinants requires a broader vision of collective wellbeing—one that reconceives security not solely as control, but as care, equity, and resilience. A decolonial approach would therefore anchor Africa’s health security in solidarity, social protection, and shared accountability, redefining the very metrics of success in continental health emergency management from rapid containment to sustainable justice and community resilience.

## Strengthening the PHECs mechanism: challenges and opportunities

### Regulatory coordination and technical governance

Despite the transformative promise of the PHECs mechanism, several challenges and tensions remain. For instance, in his declaration [25], the Africa CDC Director General urged AU Member States to fast-track emergency authorization of Mpox vaccines. As of mid-2025, over 1.01 million doses have been delivered across 12 countries, reaching 921,000 individuals, demonstrating that rapid rollout is possible where regulatory systems function [33]. Yet critical gaps remain, particularly for paediatric vaccination, as approval for children under 12 remains a challenge in many high-burden settings despite reported paediatric cases.

Although several countries—including Egypt, Ghana, Nigeria, Rwanda, Senegal, South Africa, Tanzania, and Zimbabwe—have reached Maturity Level 3 under the WHO Global Benchmarking Tool, progress across the continent remains uneven [34]. This situation therefore underscores the urgent need to fully operationalize the Africa Medicines Agency and harmonize its functions with national regulatory authorities. Strengthening AMA’s role in emergency authorizations and aligning it with Member States’ frameworks would streamline vaccine approvals, reduce bottlenecks, and ensure that the PHECs translate into rapid, actionable, and life-saving interventions.

### Decision-making processes and inclusivity

Available public information indicates that the decision to declare the PHECS was recommended by an *Emergency Consultative Group (ECG)*, of which 15 of 20 members participated [6, 25]. The group reportedly applied multiple criteria, including disease severity, transmission dynamics, health system impact, availability of vaccines and treatments, public health risk, socio-economic implications, public concern, global health security, and political considerations. The ECG has since met twice, first in February 2025 [35] and again in September 2025 [33]—to review the evolving outbreak, assess ongoing risks, and determine whether the PHECs status should be maintained. However, neither the method by which these criteria were weighted nor the decision-making procedure—whether by consensus, majority, or another mechanism—has been disclosed, limiting transparency in how the final recommendation was reached.

The process by which the 20 members were selected also remains unclear, raising questions about representativeness and inclusivity. The Consultative Group was composed primarily of technical experts (13 men and 7 women) drawn from public health and academic institutions across the continent [25]. While this composition reinforces scientific rigor, it also highlights a narrow epistemic base. The absence of structured engagement with civil society, political, and social science perspectives suggests that the process, though technically credible, remains institutionally insular. A similar configuration may be observed in the PPPR Commission that advises the AU Champion on Pandemic Prevention, Preparedness, and Response on the PHECs [25].

Such opacity mirrors long-standing criticisms of the WHO’s PHEIC process, which has faced scrutiny for its limited transparency, ambiguous accountability structures, and the disproportionate influence of the Emergency Committee in shaping outcomes. Scholars have noted that, despite the IHR’s legal prescriptions, decision-making under the PHEIC framework often departs from the stated criteria and remains subject to political negotiation as much as epidemiological assessment [8, 9, 11].

For Africa CDC, avoiding these pitfalls will be crucial. Strengthening procedural clarity in member selection, decision-making, and publication of deliberative criteria—while institutionalizing mechanisms for multidisciplinary and cross-sectoral participation—would enhance both the legitimacy and accountability of the PHECs process. By embedding transparency and inclusivity from the outset, Africa CDC can position the PHEC mechanism as a distinctively African innovation in emergency governance—technically robust, politically credible, and normatively grounded in Pan-African solidarity.

### Legal and institutional foundations

To consolidate Africa CDC’s legitimacy and ensure that the PHECs mechanism translates into effective coordination, its authority must rest on binding continental instruments rather than discretionary cooperation. The *Revised Statute of the Africa CDC* (2022), particularly *Article 25*, establishes cooperation with Member States through national focal points, technical partnerships, and requests for scientific or operational assistance [24]. Yet, these provisions remain voluntary in nature, relying on goodwill rather than obligation—insufficient for the decisive, coordinated action required during transboundary health crises.

To close this institutional gap, the AU should pursue a dedicated AU Health Protocol, modeled on the *Protocol Relating to the Establishment of the Peace and Security Council (2002)* [36]. Just as that Protocol transformed the AU’s peace and security functions from ad hoc political coordination into a legalized framework for collective action, a health protocol could codify Africa CDC’s coordinating and regulatory powers during health emergencies. This would formalize the Centre’s authority to convene Member States, direct regional responses, and trigger resource mobilization mechanisms under defined thresholds.

By embedding these powers in treaty law, the AU would align Africa CDC’s operational autonomy with Member States’ sovereignty through the principle of subsidiarity, ensuring legitimacy, predictability, and solidarity in continental health governance. In this way, the Africa CDC would evolve from a technical coordinator to a normative institution of collective health security, analogous in authority and structure to the Peace and Security Council’s role in conflict prevention.

### Financing and resource mobilization

Sustainable and predictable financing remains a critical challenge for continental health emergency responses. The Mpox outbreak exposed vulnerabilities in existing funding models, with declining foreign aid creating a gap of approximately US $220 million during the response [37]. The ECG also highlighted the withdrawal of major international support programs, including PEPFAR, leaving people living with HIV—among the most vulnerable to severe illness and death—at heightened risk [32]. These developments underscore that dependence on external aid can compromise both outbreak control and protection for high-risk populations.

The establishment of the African Epidemics Fund represents a vital step toward financial autonomy [37]; however, reliance on voluntary contributions risks inconsistencies and delays. Transparent governance, clear accountability, and predictable disbursement mechanisms are essential to maintaining confidence and avoiding politicization of resources. Linking the PHECs mechanism to continental financial institutions, such as the African Development Bank or Afreximbank, offers a pathway to rapid, reliable funding. During COVID-19, Afreximbank’s dedicated facility for vaccine procurement demonstrated the operational potential of such instruments.

Under this model, upon declaration of a PHECs and endorsement by the AU Champion on Pandemic Prevention, Preparedness, and Response, funds could be activated immediately, circumventing bureaucratic delays within AU institutions. Integrating these mechanisms with oversight processes, including lessons from successive ECG meetings, would ensure that resources are not only mobilized swiftly but are also allocated in a manner responsive to epidemiological trends and population vulnerabilities, including HIV-affected communities. This approach strengthens continental self-reliance, protects the most at-risk populations, and reinforces the continent’s capacity to lead its own emergency health response.

## Tiered activation for early response

A practical challenge also arises when an outbreak poses cross-border risk but does not meet the full threshold for a PHECs declaration. In such cases, the absence of a formal trigger may delay coordinated action. To mitigate this, Africa CDC could establish a tiered activation mechanism linked to the PHECs framework. Lower-level alerts—such as a “Regional Public Health Concern” or “Heightened Surveillance Level”—would allow conditional release of funds, technical support, and logistics coordination before a full PHECs is declared. Embedding this tiered approach within continental financial instruments would enhance early interventions while maintaining fiscal accountability.

This approach echoes the ECG September 2025 recommendation for a scalable [33] tiered alert system to maintain vigilance even as the outbreak evolves. The ECG noted that, despite improvements in testing and vaccine delivery, surges continued in Ghana, Kenya, Liberia, Zambia, and Tanzania, alongside emerging introductions in Malawi, Ethiopia, Senegal, Togo, The Gambia, and Mozambique.

While the World Health Organization lifted the PHEIC for mpox that same month [38], Africa CDC’s continued vigilance reflects the distinct epidemiological realities and operational needs within the African context. This underscores the importance of tailoring alert mechanisms to regional conditions, ensuring that decision-making remains responsive and contextually grounded.

However, global health scholars have cautioned that tiered or “traffic-light” systems can introduce new complexities and even dilute the normative force of emergency declarations. Wenham and colleagues argue that such approaches risk normalizing crises, creating mid-level “amber” alerts that fail to trigger decisive political or financial action [39]. They warn that, as seen in other sectors—including food security and counterterrorism—tiered frameworks often result in prolonged intermediate phases, data-dependent paralysis, and diminished clarity over responsibilities. In this view, the core challenge is not the absence of gradient in global alerts, but the lack of political will and compliance mechanisms to translate existing alerts into coordinated action.

Against this backdrop, a continental tiered system must differ fundamentally from the global “traffic-light” models criticized in the literature. Its success would depend on two safeguards: [1] clearly codified triggers that automatically unlock financing and technical deployment, and [2] continuous political oversight through Africa CDC’s governance bodies and AU structures. In other words, the continent’s version of tiering should be instrumental, a pragmatic tool to accelerate response, not a technical label that delays it.

### Establishing a pandemic peer review mechanism (PPRM) and multisectoral oversight

Furthermore, declining foreign aid, overlapping continental declarations or summits, and emerging global instruments such as the Pandemic Agreement highlight the urgent need for mechanisms that can sustain Pandemic Prevention, Preparedness, and Response as a lasting political and policy priority. Since the ECG first recommend the initial PHECs declaration, they have met twice to determine whether the PHECs status should be maintained. These follow-up meetings revealed operational challenges, including surges in new countries, vaccine supply and distribution gaps, surveillance deficiencies, and elevated case-fatality rates in several member states [33]. While progress has been made in areas such as testing coverage and vaccine rollout, the rapid spread of Mpox in early 2025 across the Mano River Union—a subregional organization comprising Guinea, Liberia, Sierra Leone, and Côte d’Ivoire—underscores how quickly outbreaks can escalate in the absence of coordinated, continent-wide oversight [40].

To address these vulnerabilities, Africa CDC should spearhead the creation of a **Pandemic Peer Review Mechanism (PPRM)** [41, 42], modeled on the African Peer Review Mechanism [43]. The PPRM would serve as a structured, peer-led accountability framework that complements Africa CDC’s regular PHECs briefings, independently assessing, validating, and benchmarking continental responses against established standards.

In practice, the PPRM would monitor implementation to ensure adherence to PHECs protocols, efficient mobilization of resources, and harmonization of national and continental strategies. It would also identify gaps—such as regulatory delays, logistical bottlenecks, under-resourced surveillance systems, and coordination failures—that have been repeatedly highlighted during ECG reviews. Post-PHECs assessments would generate lessons learned, guide improvements, and inform future emergency responses.

By institutionalizing such accountability, civil society and cross sectoral dialogue, the PPRM would convert the political commitments into measurable outcomes, linking results to continental and external financing mechanisms including the African Epidemics or external funding Pandemic Fund. Importantly, the synergy between Africa CDC’s weekly briefings and cross sectoral function of the PPRM would create a continuous feedback loop, pairing transparency with evidence-based oversight. This would ensure that PHECs declarations evolve from announcements into coordinated, accountable, and results-driven action (see Table 3).

**Table 3:** Strategic reforms to strengthen the PHECs mechanism and its alignment with global health governance

Domain	Strategic Reform	Purpose
Governance and coordination	Institutionalize standing coordination platforms between Africa CDC, WHO Regional Offices, and AU bodies	Ensures coherent continental–global response and prevents fragmented messaging
Emergency declaration protocols	Adopt joint, pre-negotiated criteria for emergency assessments and declarations	Minimizes conflicting signals to member states and reinforces legitimacy of regional alerts
Regulatory and data systems	Deploy African-owned interoperable surveillance and data governance frameworks	Protects data sovereignty while improving real-time risk assessment and cross-border information flow
Technical capacity and workforce	Scale joint training, field simulations, and decentralized rapid response teams	Builds sustained readiness and reduces reliance on external surge capacity
Sustainable financing	Fully operationalize the African Epidemics Fund with automatic triggers linked to PHECs activation	Guarantees predictable funds for rapid deployment and shields responses from donor volatility
Political oversight and compliance	Strengthen AU mechanisms to monitor member state adherence to PHECs directives	Enhances accountability and transforms declarations into coordinated action
Transparency and accountability	Introduce mandatory reporting and audit mechanisms for PHECs fund utilization and response metrics	Builds trust, reduces politicization, and improves response quality
Global cooperation	Formalize mutually recognized protocols with WHO to define complementary roles during emergencies	Embeds Africa CDC as a legitimate regional authority within the global health architecture

### Limitations of the CRWT framework in the context of the PHECs mechanism

While the CRWT framework provides a valuable lens for interpreting the PHECs mechanism, it has important limitations that must be considered when analyzing practical implementation. Primarily, the framework is conceptual and strategic, highlighting pathways such as Critique, Reform, Withdrawal, and Transformation, but it does not offer operational guidance for translating continental ambitions into effective health outcomes. In practice, transformative initiatives like the PHECs still face real-world constraints. For example, delays in the emergency authorization of Mpox vaccines in the Democratic Republic of Congo illustrate that even a continentally led mechanism can be hindered by gaps in national regulatory capacity and logistical bottlenecks, limiting the speed and impact of outbreak response.

Moreover, the framework does not fully capture the interdependencies and potential tensions among the pillars. Withdrawal from donor-dependent systems strengthens African autonomy, yet it must be carefully balanced with collaboration through WHO, UNICEF, and other partners to ensure operational effectiveness. The joint Mpox action plan between Africa CDC and WHO demonstrates that continental independence and global cooperation are not mutually exclusive but require careful alignment—a nuance that the CRWT framework only partially addresses.

The CRWT model also emphasizes normative and strategic intent, such as Pan-African solidarity, but it underrepresents context-specific operational challenges. The Mpox outbreak in eastern DRC, where conflict and population displacement disrupted isolation units and forced authorities to conduct searches for fleeing patients, underscores the critical role of integrating health security into the AU Peace and Security Architecture. These realities reveal that institutional mechanisms like the PHECs, AMA, Africa Epidemic Fund and PPRM must be linked with national capacities and regional security structures to achieve tangible outcomes.

Finally, the framework is primarily macro-analytical, which can obscure intra-continental variation. Differences in governance, health system maturity, and political will among AU Member States mean that the effects of a PHECs declaration are uneven.

Overall, while the framework illuminates the strategic significance of the PHECs as a decolonial initiative, it also demonstrates that aspiration alone cannot sustain transformation. Its primary value lies in guiding Africa CDC’s strategic planning toward institutional and operational maturity—by translating political vision into accountable governance, inclusive decision-making, and evidence-based implementation. In practice, this entails embedding the PHECs mechanism within predictable financing streams such as the African Epidemics Fund, strengthening regulatory harmonization through the Africa Medicines Agency, and ensuring that emergency decision-making processes are transparent, multidisciplinary, and participatory. It also requires aligning these systems with broader health, security, and governance agendas of the AU. By integrating financial sustainability, regulatory coherence, and accountable, inclusive governance, Africa CDC can move from symbolic assertion of sovereignty to a concrete model of continental health governance—one that is financially autonomous, technically capable, and politically legitimate.

## Data Availability

No datasets were generated or analysed during the current study.
